# Supplementary material for: Baby Open Brains: An Open-Source Repository of Infant Brain Segmentations
Source: bioRxiv. 2024 Oct 14:2024.10.02.616147. Preprint. [Version 2] doi: 10.1101/2024.10.02.616147 (PMC11507744; doi:10.1101/2024.10.02.616147)
Supplement: Supplement 2 [file NIHPP2024.10.02.616147v2-supplement-2.pdf]

bioRxiv preprint doi: https://doi.org/10.1101/2024.10.02.616147; this version posted October 14, 2024. The copyright holder for this preprint (which was not certified by peer review) is the author/funder, who has granted bioRxiv a license to display the preprint in perpetuity. It is made available under aCC-BY 4.0 International license.

Supplemental Table 1. No differences were seen on selected neurodevelopmental scores between the participants selected for the BOBs repository and the full BCP sample from which they were selected.

	BOBs	BCP	p-value
Mullen Early Learning Composite Score (SD)	103.09 (12.67)	104.98 (14.69)	0.34
Vineland Adaptive Behavior Composite (SD)	96.96 (7.66)	99.26 (9.26)	0.07
Infant Behavior Questionnaire - Revised: Smiling and Laughing (SD)	4.61 (0.98)	4.59 (1.10)	0.93
Infant Behavior Questionnaire - Revised: Fear (SD)	2.35 (0.82)	2.19 (0.82)	0.29
Infant Behavior Questionnaire - Revised: Duration of Orienting (SD)	3.64 (1.12)	3.65 (1.11)	0.96

^For the Vineland and IBQ-R, as differences are seen in scores across ages, BOBs participants were compared to only those in the BOBs age range. For the Mullen, the age standardized composite score was used, so BOBs participants were compared to all BCP participants. There were also no differences when just compared between those in the BOBs age range.
